# Ascorbate-Deficient *vtc2* Mutants in *Arabidopsis* Do Not Exhibit Decreased Growth

**DOI:** 10.3389/fpls.2016.01025

**Published:** 2016-07-13

**Authors:** Benson Lim, Nicholas Smirnoff, Christopher S. Cobbett, John F. Golz

**Affiliations:** ^1^School of BioSciences, University of Melbourne, ParkvilleVIC, Australia; ^2^Biosciences, College of Life and Environmental Sciences, University of ExeterExeter, UK

**Keywords:** *Arabidopsis*, ascorbate, VTC2, VTC5, vitamin C, GDP-L-galactose phosphorylase

## Abstract

In higher plants the L-galactose pathway represents the major route for ascorbate biosynthesis. The first committed step of this pathway is catalyzed by the enzyme GDP-L-galactose phosphorylase and is encoded by two paralogs in *Arabidopsis* – *VITAMIN C2* (*VTC2*) and *VTC5*. The first mutant of this enzyme, *vtc2-1*, isolated via an EMS mutagenesis screen, has approximately 20–30% of wildtype ascorbate levels and has been reported to have decreased growth under standard laboratory conditions. Here, we show that a T-DNA insertion into the *VTC2* causes a similar reduction in ascorbate levels, but does not greatly affect plant growth. Subsequent segregation analysis revealed the growth defects of *vtc2-1* mutants segregate independently of the *vtc2-1* mutation. These observations suggest that it is the presence of an independent cryptic mutation that affects growth of *vtc2-1* mutants, and not the 70–80% decrease in ascorbate levels that has been assumed in past studies.

## Introduction

Ascorbate (vitamin C) is an important multifunctional antioxidant compound involved in stress tolerance (Conklin et al., 1996; Pastori et al., 2003; Sanmartin et al., 2003; Barth et al., 2004; Filkowski et al., 2004; Müller-Moulé et al., 2004; Chen and Gallie, 2005; Larkindale et al., 2005; Pavet et al., 2005) and redox signaling (Noctor and Foyer, 2011; Page et al., 2012). While several possible biosynthetic pathways for ascorbate have been proposed, it was demonstrated that the L-galactose pathway is the dominant route in *Arabidopsis* (Dowdle et al., 2007). In this pathway, GDP-D-mannose, formed from D-mannose 1-phosphate, is sequentially converted to GDP-L-galactose, L-galactose 1-phosphate, L-galactose, L-galactono-1,4-lactone, and finally to L-ascorbate. The first committed step of this pathway is catalyzed by GDP-L-galactose phosphorylase, which is encoded by the paralogous genes *VITAMIN C2* (*VTC2*) and *VTC5* and is most likely an important control point in the pathway (Yoshimura et al., 2014).

The first ascorbate-deficient mutants of *Arabidopsis*, *vtc1-1* and *vtc2-1*, were identified via an ethyl-methanesulfonate (EMS) mutagenesis screen for ozone sensitive mutants (Conklin et al., 1996). Later, using a high-throughput nitroblue tetrazolium assay several more EMS-induced ascorbate-deficient mutants (*vtc1-2*, *vtc2-2*, *vtc2-3*, *vtc3-1*, *vtc4-1*) were identified (Conklin et al., 2000). Of these mutants, *vtc1-1* and *vtc2-1* have been extensively studied owing to their low levels of ascorbate (Veljovic-Jovanovic et al., 2001; Jander et al., 2002; Pastori et al., 2003; Müller-Moulé et al., 2004; Pavet et al., 2005; Dowdle et al., 2007; Mukherjee et al., 2010; Kempinski et al., 2011; Kerchev et al., 2011; Lee et al., 2011; Luna et al., 2011; Talla et al., 2011; Zechmann, 2011; Botanga et al., 2012; Brosché and Kangasjärvi, 2012; Page et al., 2012; Zhang et al., 2012; Wang et al., 2013). The *vtc1-1* mutant has a Pro22Ser substitution in the active site of GDP-mannose pyrophosphorylase (Conklin et al., 1999) resulting in a 70% decrease in ascorbate levels (Pavet et al., 2005; Mukherjee et al., 2010; Kerchev et al., 2011; Zechmann, 2011; Zhang et al., 2012). *vtc2-1* has a single base substitution (G to A) at the predicted 3′ splice site of the fifth intron, resulting in an 80–90% reduction in transcript levels and less than 2% of GDP-L-galactose phosphorylase activity in leaves (Dowdle et al., 2007). As a consequence, this mutant has 70–80% reduction in ascorbate levels (Müller-Moulé et al., 2004; Pavet et al., 2005; Dowdle et al., 2007; Kerchev et al., 2011). Both mutant lines display reduced growth suggesting that ascorbate plays an important role in the control of plant growth (Veljovic-Jovanovic et al., 2001; Pastori et al., 2003; Müller-Moulé et al., 2004; Pavet et al., 2005; Kerchev et al., 2011). The other *vtc2* mutants, *vtc2-*2 and *vtc2-3*, have mis-sense mutations leading to Gly223Asp and Ser290Phe substitutions, respectively (Jander et al., 2002; Dowdle et al., 2007) and while the amount of ascorbate in these lines varies according to growth conditions, values typically range from 30*–*80% and 40–50% of wildtype levels (Conklin et al., 2000; Colville and Smirnoff, 2008). Interestingly, neither *vtc2-*2 nor *vtc2-3* display noticeable growth defects (Conklin et al., 2000).

Because *vtc2-1* has detectable levels of full-length *VTC2* transcript (Dowdle et al., 2007) and is thus not a complete loss-of-function mutant, we obtained a T-DNA insertion mutant from the *Arabidopsis* stock center. We demonstrate that this mutant, here named *vtc2-4*, lacks wildtype *VTC2* transcripts and, although it has a level of ascorbate similar to *vtc2-1*, exhibits a near wildtype growth phenotype under short-day and continuous light conditions. We suggest that the decreased growth phenotype of *vtc2-1* is likely due to an independent cryptic mutation and is not due to ascorbate deficiency as widely assumed in the literature.

## Materials and Methods

### Plant Materials and Growth Conditions

Mutant alleles were all in the Col-0 background and were sourced from the ABRC stock center. For growth assays under short days, seeds were sown on soil and placed in a growth chamber and exposed to an 8 h light (150–250 μmol photons m^-2^ s^-1^)/16 h dark cycle at 21°C for 6 weeks. Plant weight was recorded and leaf surface area measurements taken by imagining freshly collected leaves and using ImageJ software to determine the surface area of leaf silhouettes. For growth under continuous light conditions, surface-sterilized seeds were germinated on agar media containing mineral salts (MM) with 2% sucrose (MMS) and 0.8% Bacto agar as described by Scholl et al. (1998). Seedlings were grown at 20°C in a climate chamber with a 16 h light (150–250 μmol photons m^-2^ s^-1^)/8 h dark cycle for 7 days before transfer to soil in a climate chamber with 24 h continuous light (150–250 μmol photons m^-2^ s^-1^).

For comparison of leaf area and chlorophyll fluorescence under short and long day conditions, plants were grown in pots (5 cm square and 5 cm deep) containing four parts Levington F2 compost (Scotts, Maryville, OH, USA) plus one part vermiculite. They were grown in short days (8 h) for 22 days after sowing in a controlled environment room at 23°C and 65% relative humidity with a light intensity of 200 μmol photons m^-2^ s^-1^. After 22 days, half the plants were transferred to long days (14 h) under otherwise identical conditions and the leaf area and chlorophyll fluorescence measured over 10 days. The plants were imaged with a CF Imager (Technologica Ltd., Colchester, UK). They were dark adapted for 30 min before measuring basal fluorescence (*F_0_*). This was followed by measuring maximum dark adapted fluorescence (*F_m_*) resulting from a saturating light flash (6, 349 μmol photons m^-2^ s^-1^ for 0.8 s). Dark-adapted quantum efficiency of photosystem II was calculated as *F_v_/F_m_* (*F_v_* = *F_m_-F_o_*) (Baker, 2008). Leaf area of the imaged rosettes was extracted from the chlorophyll fluorescence images and the instrument was calibrated with leaf discs of known area.

### Measurement of Leaf Ascorbate Content in Seedlings

Two-week old seedlings were harvested for ascorbate assay. Three seedlings from each control line (Col-0, single mutant lines) and 15–20 *vtc2-4;vtc5* seedlings were pooled for each biological replicate. Total ascorbate (ascorbate and dehydroascorbate) was measured by the iron (III) reduction assay (Kampfenkel et al., 1995) in an 8x scaled-down protocol.

### RNA Isolation and RT-PCR Analysis

Total RNA from 3-week old shoot tissues was isolated using the Qiagen RNeasy Plant Mini Kit^1^ according to the manufacturer’s protocol. The extracted RNA was further treated to remove any contaminating DNA using the DNA-*free*^TM^ kit^2^ according to the manufacturer’s protocol. First strand cDNA was synthesized using the SuperScript^®^ III First Strand Synthesis system^2^ according to the manufacturer’s protocol. The full length *VTC2* coding DNA sequence was amplified with the following primers: forward (5′-CAAAAGAGTTCCGACCGTTG-3′) and reverse (5′-ACTGAAGGACAAGGCACTCG-3′). The transcript of the constitutively expressed *ACTIN2* (*ACT2*) was used as an internal control with the following primers: forward (5′-GGTAACATTGTGCTCAGTGGTGG-3′) and reverse (5′-CTCGGCCTTGCAGATCCACATC-3′). The PCR was carried out using Promega GoTaq^®^ Green Master Mix^3^ with the following conditions: initial denaturation 5 min at 94°C was followed by 30 cycles of 30 s denaturation step at 94°C, 30 s annealing step at 60°C and 60 s extension step at 72°C. The final extension step was for 5 min at 72°C.

### Genotyping the *vtc* Mutants

Plants were genotyped by PCR using DNA extracted with the preparation method described in Edwards et al. (1991). The *vtc2-1*, *vtc5-1*, and *vtc5-2* mutants were genotyped as described by Dowdle et al. (2007). The SAIL line of *vtc2-4* was genotyped by performing PCR using a triplet of primers; two primers complementary to genomic DNA sequences situated on either side of the insertion site and a third primer complementary to the left border of the vector pDAP101. Primer sequences were as follows: forward (5′-TGATAATGGTTTCTGTAGCTTGGA-3′), reverse (5′-AAAACCAAGCTCTCTGCACAA-3′) and LB1 (5′-GCCTTTTCAGAAATGGATAAATAGCCTTGCTTCC - 3′). Accession Numbers *vtc2-4* and *vtc2-1W* (line#4) have been deposited with ABRC and given the stock numbers CS69540 and CS69541, respectively.

## Results

### Genetic Characterization of a *vtc2* T-DNA Insertion Mutant

**FIGURE 1:**
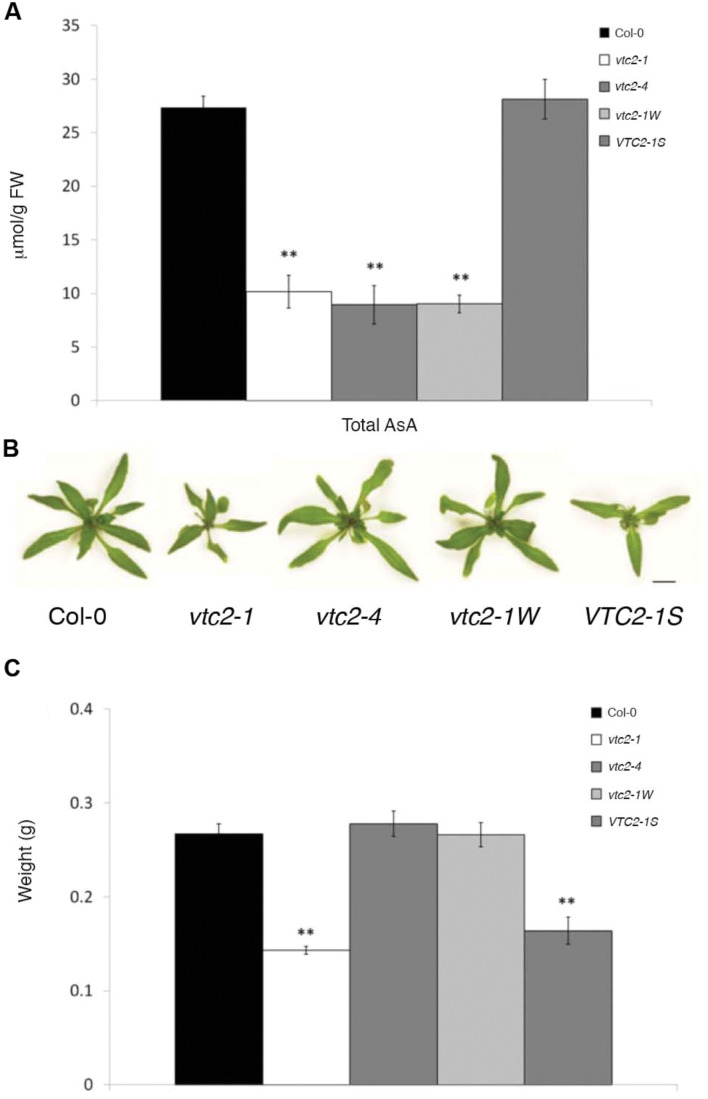
**(A–C) Leaf ascorbate concentration and fresh weights of *Arabidopsis thaliana vtc2* mutants.** **(A)** Total ascorbate (ascorbate and dehydroascorbate) concentration in leaves of 4-week-old plants. Error bars indicate SE (*n* = 3). Significant differences from Col-0 wildtype were determined with Student’s *t*-test (^∗∗^*p* < 0.01). Significance values were adjusted for multiple comparisons using the Bonferroni correction. **(B)** Three-week-old Col-0 wildtype, *vtc2-1*, *vtc2-4*, *vtc2-1W*, *VTC2-1S* grown under continuous light. Scale bar = 10 mm. **(C)** Shoot fresh weight measurements of 3-week-old plants grown under continuous light conditions. Error bars indicate SE (*n* = 6). Significant differences from Col-0 wildtype were determined with Student’s *t*-test (^∗∗^*p* < 0.01). Significance values were adjusted for multiple comparisons using the Bonferroni correction.

A line predicted to have a T-DNA insertion in *VTC2* (SAIL_769_H05/CS876707) was obtained from the *Arabidopsis* stock center (ABRC). PCR genotyping and sequencing of genomic DNA adjacent to the T-DNA left border confirmed the presence of the T-DNA within the fourth exon of *VTC2* at position +620 downstream of the start codon (+1). Lines homozygous for this insertion mutation, hereafter called *vtc2-4*, were backcrossed to wildtype (Col-0) and the resulting F2 progeny PCR genotyped for presence of the T-DNA allele. Numbers of *VTC2*/*VTC2*:*vtc2-4*/*VTC2*:*vtc2-4*/*vtc2-4* plants were consistent with an expected 1:2:1 segregation ratio (48:112:56; χ^2^ = 0.89; *p* > 0.6). Subsequent RT-PCR analysis failed to amplify a full-length *VTC2* transcript from lines homozygous for *vtc2-4* confirming presence of the T-DNA within this gene (**Supplementary Figure S1**). Measurement of total ascorbate in leaves of 4-week-old wildtype, *vtc2-1* and *vtc2-4* plants revealed that both *vtc2-4* and *vtc2-1* had approximately 30% the levels of ascorbate that are found in wildtype, showing that the T-DNA present in the *vtc2-4* line conditions a similar decrease in ascorbate (**Figure 1A**).

**FIGURE 2:**
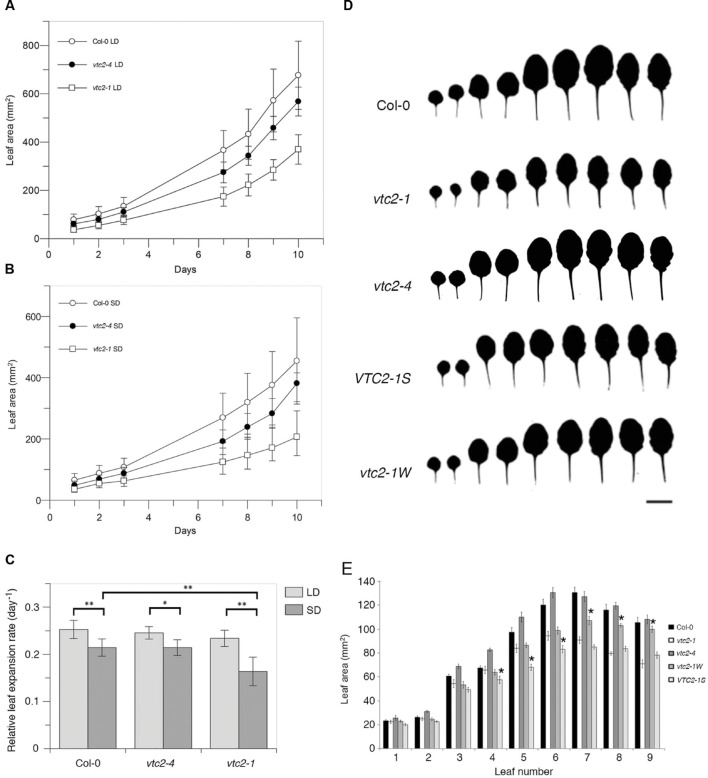
**(A–C) Rosette and leaf area measurements of *Arabidopsis thaliana vtc2* mutants.** **(A)** Surface area measurements (mm^2^) of leaves taken over a 10-day period under long-day (14 h) conditions. Values are mean and error bars indicate SD (*n* = 12). **(B)** Surface area measurements (mm^2^) of leaves taken over a 10-day period under short-day (8 h) conditions. Values are mean and error bars indicate SD (*n* = 12). **(C)** Relative leaf expansion rate (RLER, day^-1^) was calculated as the slope of a linear fit to a graph of natural log leaf area (mm) versus time (days). RLER was calculated from data in **Figures 2A,B**. Values are mean and error bars indicate SD (*n* = 10–12). Significant differences were determined with a one-way ANOVA and Tukey HSD *post hoc* test (^∗^*p* = 0.005, ^∗∗^*p* < 0.001). **(D)** Silhouettes of leaves from 6-week-old plants grown under short-day conditions. Scale bar = 1 cm. **(E)** Surface area measurements (mm^2^) of leaves taken from 6-week-old plants grown under short-day conditions. Values are mean and error bars indicate SD (*n* = 7–10). Differences between leaf areas of *vtc2-1*, *vtc2-1W* and *VTC2-1S* were assessed using a Student’s *t*-test (^∗^*p* < 0.05).

Previous studies have shown that the EMS-induced mutant *vtc2-1* has a growth defect (Müller-Moulé et al., 2004; Pavet et al., 2005; Kerchev et al., 2011). However, when *vtc2-4* mutants were propagated on soil under continuous light, they had a wildtype appearance (**Figure 1B**) and fresh weight measurements differed little from wildtype (**Figure 1C**). In contrast, *vtc2-1* mutants were significantly smaller than *vtc2-4* mutants, with approximately half the fresh weight of wildtype. To ensure that the observed growth difference between *vtc2-1* and *vtc2-4* mutants are a general feature of these mutants and not just induced by long-days, leaf surface area measurements were assessed over a 10-day growth period under both short and long-days conditions. Regardless of day length, *vtc2-4* mutants had a marginally smaller leaf surface area when compared to wildtype, whereas *vtc2-1* mutants were considerably smaller than wildtype and *vtc2-4* mutants (**Figures 2A,B**). A multi-factor ANOVA test confirmed that differences in leaf size between *vtc* mutant lines and wildtype under either growth condition was significant (*p* < 0.0001; **Table 1**). To determine whether the observed difference in leaf surface area arises from altered rates of growth, relative leaf expansion rate of rosettes under long and short day conditions were calculated. As expected, the growth rate was largely influenced by day length as each line displayed significantly higher growth under long days (**Figure 2C**). In contrast, there was no significant difference in the growth rate between wildtype and the *vtc2* mutants under long days. While this was also true for wildtype and *vtc2-4* mutants under short days, *vtc2-1* mutants had a significantly reduced growth rate when compared to wildtype (**Figure 2C**). Finding a smaller rosette leaf area for both *vtc2-1* and *vtc2-4* in long days (**Figure 2A**), but similar relative expansion rate to wildtype (**Figure 2C**), implies that the small size results from initially smaller seedlings rather than from differences in intrinsic leaf expansion rate. However, under short-day conditions, *vtc2-1* relative leaf expansion rate is compromised as well. Measuring the surface area of individual leaves collected from 6-week old short-day grown plants revealed that the size differences between wildtype and *vtc2-1* mutants is mostly confined to the fifth and subsequent leaves (**Figures 2D,E**). Taken together these data show that the growth defects of *vtc2-4* mutants are not of the same magnitude as those seen in *vtc2-1* and hence may not be directly attributable to ascorbate deficiency.

**Table 1:** A comparison of the rosette leaf areas of wildtype (Col-0) and *vtc2* mutants of *Arabidopsis thaliana* using multivariate ANOVA.

Test	Condition	*p*-value
Col-0 vs. *vtc2-4*	LD	0.0089
Col-0 vs. *vtc2-1*	LD	<0.0001
*vtc2-4 vs. vtc2-1*	LD	<0.0001
Col-0 vs. *vtc2-4*	SD	<0.0001
Col-0 vs. *vtc2-1*	SD	<0.0001
*vtc2-4 vs. vtc2-1*	SD	<0.0001


*The leaf area data are shown in **Figures 2A,B.***

### The Small Size Phenotype of *vtc2-1* Is Not Linked to Ascorbate Deficiency

Since *vtc2-1* and *vtc2-4* have contrasting growth phenotypes, we undertook a genetic characterization of *vtc2-1*. From a cross between wildtype and *vtc2-1* F1 individuals, heterozygous for *vtc2-1*, exhibited wildtype growth in soil under continuous light condition (data not shown). The presence of the *vtc2-1* allele can be detected by CAPS (cleaved amplified polymorphic sequence) PCR (Dowdle et al., 2007). In a segregating F2 population grown in soil, genotyping confirmed that the *vtc2-1* allele segregated in a Mendelian ratio (**Table 2**; χ^2^ = 2.23; *p* > 0.3). Four-week-old individual F2 plants were also visually scored for size as “Wildtype” or “Small.” While wildtype and *vtc2-1* mutants grown in parallel showed a clear difference in size as previously reported (Veljovic-Jovanovic et al., 2001; Müller-Moulé et al., 2004; Pavet et al., 2005; Kerchev et al., 2011), some individual wildtype plants exhibited a similar growth size to *vtc2-1* mutant plants. However, an excess of small individuals over the expected number suggested it was likely that some wildtype individuals were misclassified as “Small.” A number of individuals with a small growth phenotype were homozygous for the wildtype *VTC2* allele and, conversely, some *vtc2-1* homozygous individuals had a wildtype appearance (**Table 2**).

**Table 2:** Phenotypic scoring of 4-week-old F2 individuals from a cross between wildtype (Col-0) and *vtc2-1 Arabidopsis thaliana* mutants.

Phenotype	*VTC2*/*VTC2*	*VTC2*/*vtc2-1*	*vtc2-1*/*vtc2-1*	Total
Small	12	34	44	90
Wildtype	60	126	31	217
Total	72	160	75	


To score the growth phenotype more accurately a number of F2 lines derived from the Col-0/*vtc2-1* cross were allowed to self-fertilize to produce individual F3 families, which were then observed as populations after 4-week-growth in soil under continuous light conditions (**Table 3**). Of seven F2 individuals wildtype at the *VTC2* locus with an apparent small growth phenotype, five F3 families were uniformly small in size (called *VTC2-1S;* **Figure 1B**). Conversely, of 24 F2 individuals homozygous for *vtc2-1* and apparently wildtype in size, 18 F3 families were uniformly wildtype in size (called *vtc2-1W*; **Figure 1B**). Measuring fresh weight and total ascorbate levels under continuous light conditions revealed that *vtc2-1W* plants had a similar weight to wildtype, but with ascorbate levels decreased by approximately 70% compared to wildtype. Likewise, fresh weight of a representative *VTC2-1S* line was similar to *vtc2-1*, but with ascorbate approaching wildtype levels (**Figure 1C**).

**Table 3:** Growth phenotypes of 4-week-old F3 individuals.

Phenotype and genotype of F2 line	Growth phenotypes segregating	Growth phenotypes uniform
Small – *VTC2*/*VTC2 * (*VTC2-1S*)	2	5^a^
Wildtype – *vtc2*/*vtc2 * (*vtc2-1W*)	6	18^b^


*Seeds derived from F2 lines were germinated on agar media under long day conditions for 7 days before transfer to soil under continuous light. Growth phenotype of 4-week-old F3 individuals were scored relative to Col-0 and *vtc2-1* plants grown in parallel.*

*^a^All plants have a small growth phenotype. ^b^All plants have a wildtype growth phenotype.*

To better characterize the growth characteristics of these F3 lines, fresh weight and leaf surface area measurements of representative *vtc2-1W* and *VTC2-1S* plants grown under short-day were made (**Figures 2D,E**, **Supplementary Figure S2**). In agreement with the measurements made under continuous light conditions, the fresh weight of the *vtc2-1W* line was statistically different from *vtc2-1* mutant controls (**Supplementary Figure S2**). When compared to *vtc2-1* mutants, late arising leaves of *vtc2-1W* plants were larger (leaf 7 to leaf 9; increase ranged from 18 to 41%). In contrast, the fresh weight of a representative *VTC2-1S* line was not significantly different from *vtc2-1* mutants (**Supplementary Figure S2**). This is despite finding that a few *VTC2-1S* leaves were slightly smaller than those of *vtc2-1* (leaf 5–6; decrease ranged from 13 to 23%). In summary, these analyses demonstrate that ascorbate-deficiency is genetically separable from growth defects associated with the *vtc2-1* mutant line.

### Leaf Senescence in *vtc2* Mutants

**FIGURE 3:**
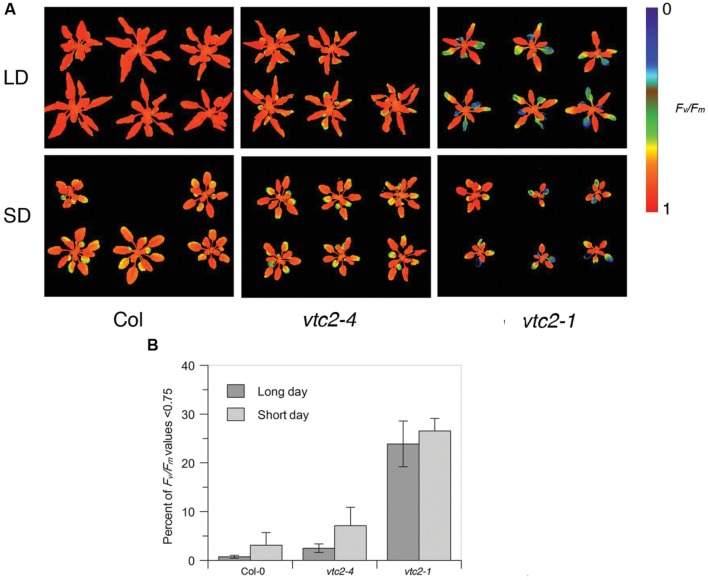
**(A,B) *F_v_/F_m_* measurements of *Arabidopsis thaliana vtc2* mutants.** **(A)** Chlorophyll fluorescence images of short-day (SD) and long-day (LD) grown plants colored according to their *F_v_/F_m_* ratio on day 10 (see **Figures 2A,B**). **(B)** Percentage of leaf surface area with *F_v_/F_m_* values below 0.75 in short-day and long-day grown plants. Data are pooled from days 7–10 (see **Figures 2A,B**). Error bars indicate SD (*n* = 6–12). ANOVA shows significant differences (*p* < 0.001) between Col-0 and the *vtc2-1* mutant, whereas daylength only has a significant effect on *vtc2-4* mutant plants (*p* < 0.05).

Given that ascorbate deficiency in *vtc2-4* mutants is not associated with a strong growth defect, we next considered whether this line displays other ascorbate-deficient characte ristics. Previous studies have shown that reduced ascorbate is associated with faster leaf senescence (Barth et al., 2004). Senescence can be measured as a loss of leaf photosynthetic efficiency. Thus to assess the extent of leaf senescence in *vtc2* mutants, we imaged dark-adapted quantum efficiency of photosystem II (*F_v_/F_m_*). Examining the *F_v_/F_m_* images of wildtype, *vtc2-4* and *vtc2-1* grown under both short and long days suggested bigger decreases in older leaves of *vtc2* mutants compared to wildtype (**Figure 3A**). To quantify the different rates of senescence in each line, the % of leaf area below a fixed *F_v_/F_m_* value (0.75) over days 7–10 was calculated. This revealed a slight elevation in the rate of senescence under short days compared to long days for all lines tested which was only significant for *vtc2-4* (*p* < 0.05) (**Figure 3B**). However, under both growth conditions, *vtc2-1* mutants displayed a noticeable and statistically significant (*p* < 0.001) increase in the proportion of *F_v_/F_m_* values below 0.75 when compared to wildtype, whereas *vtc2-4* mutants only exhibit a marginal and statistically non-significant increase (**Figure 3B**). These observations are consistent with *vtc2-1* mutants displaying increased leaf senescence, which is not apparent in *vtc2-4* under these growth conditions.

### *Arabidopsis* Lacking Functional GDP-L-galactose Phosphorylase has Undetectable Ascorbate and Exhibits a Seedling-Lethal Phenotype

**FIGURE 4:**
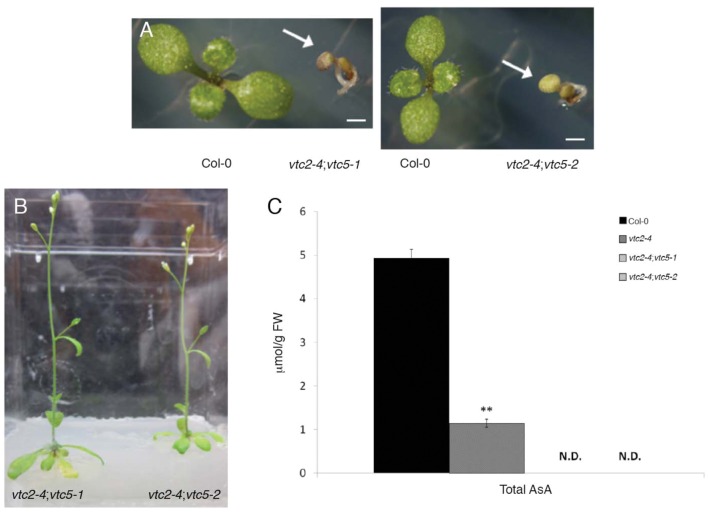
**(A–C)** Growth and leaf ascorbate concentration of *Arabidopsis thaliana vtc2-4;vtc5* double mutants. **(A)** One-week old Col-0 and *vtc2-4;vtc5-1* double mutants (arrow) grown on agar media. Bar = 1 mm. **(B)** Three-week-old *vtc2-4;vtc5-1* double mutants grown on agar media supplemented with 0.5 mM L-ascorbic acid. **(C)** Total ascorbate (ascorbate and dehydroascorbate) concentration in leaves of 2-week-old seedlings grown on agar medium. Three seedlings from Col-0 and *vtc2-4* and 15–20 *vtc2-4;vtc5* seedlings were pooled for each sample. Error bars indicate SE (*n* = 3). Significant differences from Col-0 wildtype were determined with Student’s *t*-test with significance values adjusted for multiple comparisons using the Bonferroni correction (^∗∗^*p* < 0.01).

The importance of the L-ascorbate biosynthetic pathway for *Arabidopsis* growth and development was demonstrated by Dowdle et al. (2007) when they showed that *vtc2-1*;*vtc5* double mutants displayed a growth arrest following germination; a phenotype that was also associated with cotyledon bleaching. Given that *vtc2-1* is not a complete loss-of-function allele and also apparently harbors a cryptic mutation/s that affect growth, we reconstituted the *vtc2;vtc5* double mutant line by combining the *vtc2-4* mutant with two different *vtc5* mutant alleles (*vtc5-1* and *vtc5-2*; Dowdle et al., 2007). The resulting *vtc2-4;vtc5-1* and *vtc2-4;vtc5-2* double mutants also exhibited a seedling-lethal phenotype, and cotyledons were subsequently bleached upon germination (**Figure 4A**). These were phenotypically indistinguishable from the *vtc2-1;vtc5* double mutants previously described (Dowdle et al., 2007). Consistent with the previous study, both double mutants had undetectable levels of ascorbate (**Figure 4B**). The double mutants could be rescued to complete their life cycles by supplementation with ascorbate (**Figure 4C**). Thus these experiments show that the phenotype of the double mutants between *vtc2-1* and *vtc5* produced by Dowdle et al. (2007) is not caused by additional mutations in *vtc2-1* and confirm the importance of the L-ascorbate biosynthetic pathway for seedling viability.

## Discussion

It has been widely reported that *Arabidopsis* mutants with severe ascorbate deficiency exhibit a small growth phenotype and that this decreased growth is thus casually linked to ascorbate deficiency (Jander et al., 2002; Müller-Moulé et al., 2004; Pavet et al., 2005; Dowdle et al., 2007; Giacomelli et al., 2007; Kerchev et al., 2011). Here we show that the decreased growth phenotype of the *vtc2-1* mutant line is not linked to ascorbate deficiency using two approaches; (1) the *vtc2-4* mutant, an apparent null mutant which has comparable levels of ascorbate to *vtc2-1*, shows a different growth profile when compared to *vtc2-1* mutants; (2) after backcrossing *vtc2-1* to wildtype plants we readily isolated *vtc2-1* ascorbate-deficient lines with weight and leaf sizes that are closer to wildtype than *vtc2-1* mutants, as well as lines with a small growth phenotype that were homozygous for *VTC2*. It appears that the decreased growth phenotype of the *vtc2-1* line is caused by an additional mutation/s that have likely arisen during the original EMS mutagenesis process (Conklin et al., 1996). Because of the difficulty of accurately scoring the size of individual F2 plants it is not clear from our analysis whether the *vtc2* mutation and those associated with the growth defect are linked. However, it is unlikely that they are closely linked because it was relatively easy to identify F2 individuals homozygous for the wildtype allele at one locus but homozygous mutant at the other.

The *vtc1-1* mutant (Conklin et al., 1996) also exhibits a decreased growth phenotype (Veljovic-Jovanovic et al., 2001; Pavet et al., 2005; Kerchev et al., 2011; Talla et al., 2011). VTC1 produces GDP-mannose, which has important functions in cell wall carbohydrate biosynthesis and protein glycosylation in addition to ascorbate biosynthesis (Conklin et al., 1999; Lukowitz et al., 2001). It is therefore possible that deficiency of GDP-mannose results in a number of different biochemical defects leading to a small growth phenotype that is not specifically due to ascorbate deficiency.

The *vtc2-1* mutant line has been extensively analyzed (Müller-Moulé et al., 2003; Mukherjee et al., 2010; Gao et al., 2011; Zechmann, 2011; Botanga et al., 2012; Page et al., 2012) and has also been used to generate different mutant combinations in a number of studies. For example, interactions between redox metabolism and abscisic acid signaling were investigated by crossing *vtc2-1* with an abscisic acid insensitive mutant (*abi4*). The *vtc2-1;abi4* double mutant that was subsequently characterized, exhibited wildtype growth leading to the suggestion that *abi4* suppressed the growth phenotype of *vtc2-1* (Kerchev et al., 2011). It is plausible that from the cross, the *vtc2-1;abi4* double mutant selected for analysis lacked the cryptic mutation(s) conferring the small growth phenotype. Furthermore, the use of whole genome microarray analysis to characterize differences between *vtc2-1* and *vtc2-1 abi4* mutant lines is likely to have been complicated by the possible absence of the cryptic mutation(s) in the double mutant background (Kerchev et al., 2011). The decreased growth phenotype of *vtc2-1* has also been described in combination with mutations affecting chronic photo-oxidative stress (Müller-Moulé et al., 2004), chloroplastic ascorbate peroxidase (Giacomelli et al., 2007) and the autoimmune response (Zhu et al., 2013).

Our data indicate that the ascorbate deficiency of *vtc2* mutants does not cause a large decrease in growth under laboratory conditions. While the *vtc2-4* has a slightly smaller rosette area than wildtype, relative leaf expansion rate is identical. It is likely that *vtc2-4* seedlings have smaller initial seedling size or vigor resulting in a magnifying size difference as the plants grow. Since the fully ascorbate deficient seedlings of the *vtc2;vtc5* double mutant are not viable, it is apparent that a reduction greater than the ∼80% occurring in the *vtc2-4* mutant is needed to impact growth. Ascorbate is amongst the most abundant of primary metabolites in *Arabidopsis* leaves (compare the widely reported values of 2–20 μmol g^-1^ fresh weight for ascorbate with the most abundant sugars, organic acids and amino acids reported in Szecowka et al., 2013). The presence of ascorbate concentrations in leaves that are much greater than needed to support growth in laboratory conditions emphasizes the importance of its protective functions for plants growing in stressful or fluctuating natural environments (Munné-Bosch et al., 2013).

## Conclusion

In summary, we suggest that the interpretation of some studies of the commonly used *vtc2-1* line or its derivatives may need to be re-evaluated. It is also important to take into consideration that the apparent cryptic mutation(s) affecting growth of *vtc2-1* may have a significant impact on physiological status and gene expression unrelated to ascorbate deficiency, so although it was critical in identifying its biosynthetic pathway, it is not suitable for investigating the wider functions of ascorbate.

## Author Contributions

BL, NS, and JG designed and performed the experiments, analyzed the data and drafted the manuscript. CC conceived the study and its design and coordination, and assisted with revisions of the manuscript. All authors read and consented to the final version of the manuscript.

## Conflict of Interest Statement

The authors declare that the research was conducted in the absence of any commercial or financial relationships that could be construed as a potential conflict of interest.

## Footnotes

1 www.qiagen.com

2 www.lifetechnologies.com

3 www.promega.com

## Supplementary Material

**FIGURE S1 | Reverse transcription polymerase chain reaction analysis of the *vtc2-4* mutant.** Total RNA was extracted from 10-day old seedlings and RT-PCR was performed using *VTC2* specific primers (top panel) that flank the full-length transcript. *ACTIN2* (*ACT2*) specific primers were used as an internal control (bottom panel).

**FIGURE S2 | Fresh weight measurements of short-day grown plants.** Shoot fresh weights of indicated lines were assessed after 6-week growth under short-day conditions. Error bars indicate SE (*n* = 7–10). Significant differences were determined using a Student’s *t*-test and statistical differences (*p* < 0.01) from Col-0 wildtype indicated with “a,” whereas “b” denotes differences from *vtc2-1* mutants.

## Abbreviations

ABRC: Arabidopsis Biological Resource Centre
EMS: Ethyl-Methanesulfonate
RT-PCR: Reverse Transcription Polymerase Chain Reaction
SAIL: Syngenta Arabidopsis Insertion Library
