# Supplementary figures and images for: Ascorbate-Deficient vtc2 Mutants in Arabidopsis Do Not Exhibit Decreased Growth
Source: Front Plant Sci. 2016 Jul 13;7:1025. doi: 10.3389/fpls.2016.01025 (PMC4943039; doi:10.3389/fpls.2016.01025)

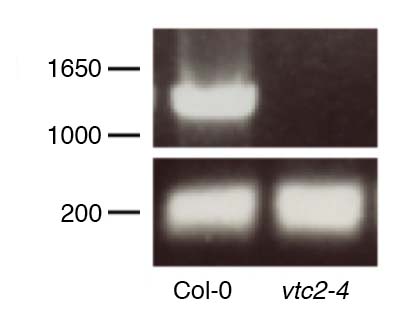

Supplement: Supplementary file 1 [file Image_1.JPEG]

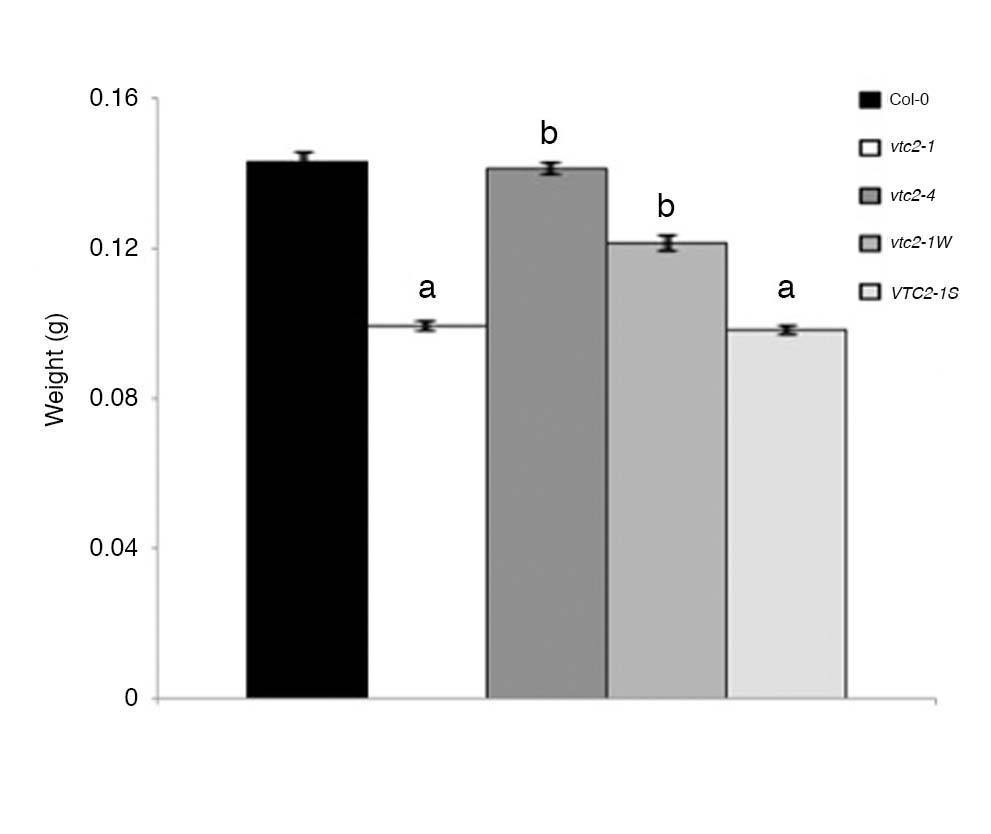

Supplement: Supplementary file 2 [file Image_2.JPEG]
